# Changing Brain Networks Through Non-invasive Neuromodulation

**DOI:** 10.3389/fnhum.2018.00128

**Published:** 2018-04-13

**Authors:** Wing Ting To, Dirk De Ridder, John Hart Jr., Sven Vanneste

**Affiliations:** ^1^School of Behavioral and Brain Sciences, The University of Texas at Dallas, Richardson, TX, United States; ^2^Department of Surgical Sciences, Section of Neurosurgery, Dunedin School of Medicine, University of Otago, Dunedin, New Zealand

**Keywords:** transcranial magnetic stimulation, transcranial direct current stimulation, stimulation target, brain hubs, brain networks

## Abstract

**Background/Objective**: Non-invasive neuromodulation techniques, such as repetitive Transcranial Magnetic Stimulation (rTMS) and transcranial Direct Current Stimulation (tDCS), have increasingly been investigated for their potential as treatments for neurological and psychiatric disorders. Despite widespread dissemination of these techniques, the underlying therapeutic mechanisms and the ideal stimulation site for a given disorder remain unknown. Increasing evidence support the possibility of non-invasive neuromodulation affecting a brain network rather than just the local stimulation target. In this article, we present evidence in a clinical setting to support the idea that non-invasive neuromodulation changes brain networks.

**Method**: This article addresses the idea that non-invasive neuromodulation modulates brain networks, rather than just the local stimulation target, using neuromodulation studies in tinnitus and major depression as examples. We present studies that support this hypothesis from different perspectives.

**Main Results/Conclusion**: Studies stimulating the same brain region, such as the dorsolateral prefrontal cortex (DLPFC), have shown to be effective for several disorders and studies using different stimulation sites for the same disorder have shown similar results. These findings, as well as results from studies investigating brain network connectivity on both macro and micro levels, suggest that non-invasive neuromodulation affects a brain network rather than just the local stimulation site targeted. We propose that non-invasive neuromodulation should be approached from a network perspective and emphasize the therapeutic potential of this approach through the modulation of targeted brain networks.

## Introduction

The use of invasive and non-invasive neuromodulation for the treatment of neurological and psychiatric disorders has grown exponentially in recent years, with increasing interest in non-invasive neuromodulation. In neurorehabilitation, non-invasive neuromodulation techniques, such as repetitive Transcranial Magnetic Stimulation (rTMS) and transcranial Direct Current Stimulation (tDCS), have been proven to ameliorate symptoms in depressive disorders, pain, aphasia, movement disorders, motor stroke, multiple sclerosis, epilepsy, disorders of consciousness, Alzheimer’s disease, tinnitus, schizophrenia, substance abuse, addiction and craving, amongst others (e.g., Fregni et al., 2008; Benninger et al., 2010; Van den Eynde et al., 2010; Zyss, 2010; Freitas et al., 2011; Avenanti et al., 2012; Borckardt et al., 2012; Brunelin et al., 2012; Brunoni et al., 2012; Sun et al., 2012; Berlim et al., 2013; Li et al., 2013; Marangolo et al., 2013; Villamar et al., 2013b; Donnell et al., 2015; Douglas et al., 2015; Shekhawat et al., 2016).

RTMS and tDCS are assumed to induce neuroplastic changes through the application of magnetic or electrical stimuli, respectively, directly to a brain area. For rTMS, brief high-current pulses are produced in a coil of wire, called the magnetic coil (Shapira-Lichter et al., 2013). The magnetic field is produced with the line of flux passing perpendicular to the plane of the coil, which is usually placed tangentially to the scalp (Shapira-Lichter et al., 2013). RTMS modulates cortical excitability using either inhibitory, low-frequency (≤1 Hz) or facilitatory high-frequency (≥5 Hz) stimulation. Furthermore, there are recently developed rTMS paradigms aimed at modifying cortical excitability, such as theta burst stimulation (TBS), delivered as a continuous (i.e., cTBS) or an intermittent theta burst stimulation (i.e., iTBS) train. cTBS is suggested to be similar to 1 Hz-rTMS, mimicking the long-term depression (LTD) of synaptic plasticity, whereas iTBS is suggested to be excitatory (Di Lazzaro et al., 2005; Huang et al., 2005; Noh et al., 2015). More specifically for cTBS, Ji et al. (2017) have demonstrated that low frequency (LF; 1 Hz) rTMS and cTBS share similar short-term aftereffects in both topographical and temporal profiles, suggesting a similar underlying mechanism (Ji et al., 2017). TDCS influences brain excitability by using a low level of continuous electrical current. For tDCS, two (or more) electrodes are placed on the scalp with the current going from the anode to the cathode. As opposed to rTMS, the electrical currents delivered by tDCS are not strong enough to fire an action potential (Radman et al., 2009). The actual plastic effects of both techniques, however, are also dependent on the state of the stimulated cortex. It has been suggested that the stimulation can potentially interact with the prior state of the cortex (Filmer et al., 2014). Several factors can influence excitatory/inhibitory changes of brain stimulations (Filmer et al., 2014), including the state of the brain during stimulation (at rest or paired with a task; Horvath et al., 2014), any intake of substances such as nicotine (Thirugnanasambandam et al., 2011) and even the time of the day (Sale et al., 2007). This sliding of the modification threshold for increased excitation (or long-term potentiation, LTP) and decreased excitation (or LTD), depending on the previous history of neural activity is referred as “metaplasticity” or “homeostatic plasticity” (Abraham and Bear, 1996; Desai, 2003; Lang et al., 2004; Siebner et al., 2004; Abraham, 2008; Cosentino et al., 2012; Hulme et al., 2013; Bocci et al., 2014).

Currently, behavioral manifestations of neurological and psychiatric diseases are seen as a result of alterations in a brain network and its connectivity as opposed to an abnormality in one isolated brain region (Fox et al., 2012b; Shafi et al., 2012; Luft et al., 2014; Fornito and Bullmore, 2015). Neuroscientific research has shifted focus from the properties of individual brain regions to the interactions and connections between brain regions (Fox et al., 2012b). Even in a recent reappraisal of historic cases, such as Phineas Gage, Louis Victor Leborgne, and Henry Gustave Molaison, researchers indicated that the disruptions extended to areas far from the site of the lesions themselves (Thiebaut de Schotten et al., 2015).

Neuromodulation research has also shifted its approach from targeting individual brain regions to targeting entire brain networks (Sale et al., 2015). Increasing evidence points to the influence of neuromodulation on whole brain networks by stimulating just one brain region (or brain hub; Grefkes and Fink, 2011; Fox et al., 2012b). Furthermore, the positive clinical effects of non-invasive neuromodulation in various disorders are presumably caused by the complex interactions between the associated brain network and the stimulation target (Kunze et al., 2016). Interestingly, studies stimulating one important brain hub involved in different processes and disorders, such as the dorsolateral prefrontal cortex (DLPFC), have shown to be effective for several conditions, which is expected since this core hub is involved in general cognitive and emotional processing. On the other hand, studies using different target locations (i.e., various brain areas in the altered brain network) for the same disorder have shown to have similar results, as in the case of tinnitus and major depression. Evidence for the effectiveness of different stimulation targets for one neuropathology as well as evidence for the effectiveness of one stimulation target for different pathologies may indicate an underlying neural network for disorders and may consequently suggest network stimulation as a new stimulation protocol.

In this review we discuss non-invasive neuromodulation techniques, namely rTMS and tDCS and their possible effects on functional connectivity in the brain. We briefly describe the basic brain network models and their hubs. Then, we pursue confirmation for possible functional network effects of rTMS and tDCS across different levels of the central nervous systems, i.e., a whole-brain functional connectivity level as well as a neurometabolite concentration level. Lastly, we present further evidence in clinical studies demonstrating possible effects on the functional connectivity of the brain for rTMS and tDCS on different stimulation sites and different disorders by: (1) presenting various effective stimulation sites for one disorder; and (2) presenting one effective stimulation site for different disorders.

## Brain Networks and Their Hubs

**Figure 1:**
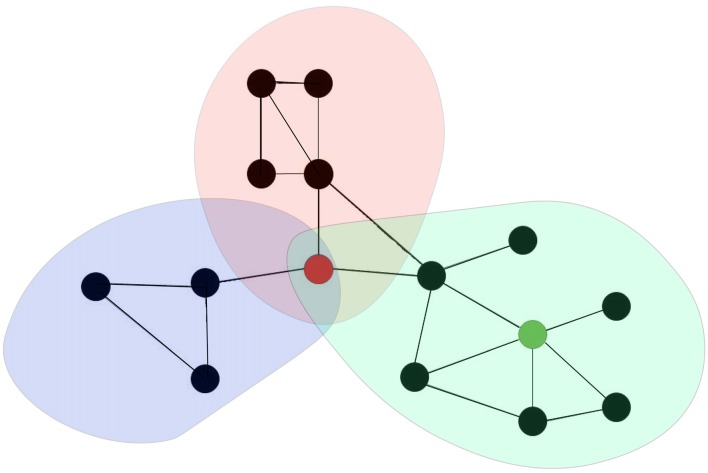
Brain networks can be represented as a graph comprising of a set of nodes (black dots) and a collection of edges (black lines in between the black dots). A subset of nodes of the network that show strong interaction among each other than other nodes in other subset of nodes are represented as modules (colored communities). Provincial hubs are highly connected nodes that primary connect nodes in the same module (e.g., green node). Connector hubs are highly connected nodes that has a diverse connectivity profile because it is connecting to several different modules within the brain network (e.g., red node).

The human brain is a complex network of interlinked regions (van den Heuvel and Sporns, 2011; Fornito et al., 2013). This network can be mathematically represented as graphs, comprised of nodes (neuronal elements) and edges (their connections) that describe the brain’s structural connectivity (Fornito et al., 2013; van den Heuvel and Sporns, 2013a). A subset of nodes (brain regions) with strong internal interaction and relatively weak external associations are represented as modules (van den Heuvel and Sporns, 2013b; Cocchi et al., 2015). Communication between modules is supported by brain hubs, which are regions that are densly interconnecteds with other brain areas and play an important role in the integration of information between different parts of the network (van den Heuvel and Sporns, 2011; Cocchi et al., 2015; see Figure 1). In addition to the classification of hubs on the basis of anatomical or structural connectivity, numerous studies have also investigated the existence of “functional hubs” derived from networks of dynamic interactions between brain regions (van den Heuvel and Sporns, 2013a). Connectivity in brain networks can indeed be assessed in different ways (Friston, 2011). Anatomical or structural connectivity, the stable direct physical pathways linking spatially distinct brain areas, is distinguished from dynamic functional connectivity and effective connectivity (Friston, 2011; Shafi et al., 2012; Fornito et al., 2013; Luft et al., 2014). Effective connectivity refers to the directional flow of information or the causal relationships between (Friston, 2011; Shafi et al., 2012; Fornito et al., 2013; Luft et al., 2014) nodes, whereas functional connectivity simply computes measures of statistical independence (correlation) between nodes (Friston, 2011; Shafi et al., 2012; Fornito et al., 2013; Luft et al., 2014).

Conceptualizing the brain as a network (Sporns et al., 2005) might have important implications for understanding clinical brain disorders (Bullmore and Sporns, 2012; Menon, 2013; Rubinov and Bullmore, 2013; van den Heuvel and Sporns, 2013a; Crossley et al., 2014). A generic property of a network is that dysfunction can spread easily between linked elements, leading to pathological cascades that include large parts of the system (Buldyrev et al., 2010; Huang et al., 2011). Fornito et al. (2015) have proposed different versions of a maladaptive response that can mediate the spread of pathology throughout the connectome, as well as the resources and processes that enable adaptation. Studies have suggested that damage to “provincial” hubs—those that are the primary link to other nodes in the same module and have an important role in functional specialization—will lead to specific clinical deficits, whereas damage to “connector” hubs—those that have links that are distributed across multiple different modules and have a central role in functional integration—will result in more complex and pervasive dysfunction and is proposed to impair multiple behavioral domains (Fornito et al., 2015). In general, it has been suggested that brain hubs are indeed involved in the anatomy of various brain disorders (Crossley et al., 2014). Studies have further found extensive anatomical overlap between structural abnormalities or lesions in various brain disorders (Crossley et al., 2014), with a similar result reported in another study of a subgroup of psychiatric disorders (Goodkind et al., 2015). For example, a recent meta-analysis of structural and functional neuroimaging studies found a collective core of brain regions affected by most psychiatric disorders, centered on dorsal anterior cingulate cortex (dACC) and the insula (Goodkind et al., 2015; Downar et al., 2016). These nodes correspond to an anterior cingulo-insular or “salience” network and are suggested to stand at a crossroads within the functional architecture of the brain, acting as a switch to deploy other major functional networks according to motivational demands and environmental constraints (Downar et al., 2016). The authors proposed these regions as promising targets for brain stimulation in psychiatric disorders. With more advanced designs of non-invasive neuromodulation, studies have shown the possibility of targeting these deeper brain structures using, for example, double cone coil rTMS (Hayward et al., 2007; De Ridder et al., 2011; Deng et al., 2014; Kreuzer et al., 2015) or high definition tDCS (DaSilva et al., 2015).

## Influence of Non-invasive Neuromodulation in Functional Brain Networks

The effects of non-invasive neuromodulation on network-based connectivity can be observed and investigated across different levels of the central nervous system, such as a whole-brain functional connectivity level as well as a neurometabolite concentration level. In this section, studies adopting both a “macro” as well as a “micro” perspective of the brain’s neural structure will be examined to understand the distributed processing of neuromodulation in functional brain networks.

### MACRO-Level: Assessing Network Connectivity With Task-Independent and Task-Related Neuroimaging

Recent studies have shown that non-invasive neuromodulation techniques affect brain connectivity patterns both while performing tasks and while at rest (Grefkes et al., 2010; Alon et al., 2011; Keeser et al., 2011a; Polanía et al., 2011a, 2012; Vanneste and De Ridder, 2011; Meinzer et al., 2012, 2013; Peña-Gómez et al., 2012; Chib et al., 2013; Park et al., 2013; Stagg et al., 2013; Ding et al., 2014; Weber et al., 2014), suggesting that stimulation is not influencing the target area in isolation, but rather a brain network (Alon et al., 2011). These techniques enable the perturbation of large-scale neural systems and may facilitate in testing whole brain models (Breakspear, 2017). Most studies examined the effects of tDCS or rTMS on brain connectivity by analyzing changes in functional networks during task-independent (resting-state) or task-related neuroimaging (e.g., EEG, fMRI). Therefore, in this article, only studies combining neuroimaging with tDCS and rTMS will be presented.

Task-independent or resting-state neuroimaging studies have investigated the effect of tDCS or rTMS on short term reorganization in functional networks in healthy subjects (for review see Luft et al., 2014) and in patients (e.g., tinnitus patients, Vanneste and De Ridder, 2011) using fMRI (for review see Luft et al., 2014) or EEG (Polanía et al., 2011a; Vanneste and De Ridder, 2011). The studies varied in how they defined the nodes of the network (Luft et al., 2014). Most studies identified specific regions of interest to look into brain connectivity.

For tDCS studies, the specific brain regions of interest varied from M1 (Polanía et al., 2011a,b, 2012) to DLPFC (Vanneste and De Ridder, 2011; Park et al., 2013; Stagg et al., 2013; Weber et al., 2014). These studies all combined tDCS with either resting-state EEG or resting-state fMRI to examine for example whether tDCS induces increased or decreased functional connectivity between specific brain areas (e.g., intrahemispheric or interhemispheric connectivity changes; e.g., Polanía et al., 2011b; Sehm et al., 2013) or whether tDCS modulates functional connectivity of certain circuits (e.g., Alon et al., 2011; Polanía et al., 2012) in a non-clinical population. For instance, studies using tDCS to target the left DLPFC in healthy subjects have been shown to influence regional electrical activity in both surface level and deeper structures using resting-state EEG (Keeser et al., 2011b) and resting-state fMRI (Keeser et al., 2011a; Peña-Gómez et al., 2012; Park et al., 2013; Stagg et al., 2013). Keeser et al. (2011b) used resting-state EEG and sLORETA to demonstrate that tDCS on the left DLPFC influences electrical activity in both during surface areas measured by EEG and deeper structures in the prefrontal lobe, such as the medial frontal gyrus, the anterior cingulate cortex and the subgenual anterior cingulate cortex. They further suggested that tDCS might influence this whole network during resting state, making it easier to activate the network during consecutive cognitive task performances, as a positive impact of tDCS was seen on a following n-back task (on error rate, accuracy and reaction time). Park et al. (2013) used resting-state fMRI to demonstrate that tDCS on the left DLPFC increased interhemispheric connectivity, suggesting that this mechanism may enhance cognitive functioning. Using resting-state fMRI, Keeser et al. (2011a) further assessed resting-state connectivity after left DLPFC and found significant changes in the default mode network (DMN) and the right and left frontal parietal network (FPN), both close to the primary stimulation site and in connected brain areas. In addition, Peña-Gómez et al. (2012) revealed increased synchrony in the anticorrelated network—a network of brain areas revealing strong negative activity correlation with the DMN and associated with cognitive processing—components and reduced synchrony in the DMN after tDCS over the left DLFPC, assessed with resting-state fMRI. This finding suggests that tDCS over the DLPFC may enhance the flexible balance between brain networks by enhancing network connectivity for cognitive demands while reducing DMN activity. Interestingly, Stagg et al. (2013) focused on brain perfusion changes during left DLPFC tDCS (using whole-brain arterial spin labeling) and found decreased functional connectivity between the left DLPFC and the bilateral thalami after tDCS, possibly offering a mechanistic explanation for the analgesic effects of tDCS in pain studies while adding weight to the hypothesis that the DLPFC modulates pain via a decrease in thalamic activity. In patient populations, one study in tinnitus patients, combining tDCS with resting-state EEG, had demonstrated that bifrontal DLPFC tDCS was able to suppress tinnitus by modulating the pregenual anterior cingulate cortex, the parahippocampal area, and the right primary auditory cortex in resting-state spontaneous brain activity (Vanneste and De Ridder, 2011). This study is the first conducted in a patient population to provide support that tDCS has an impact not only on the underlying DLPFC, but also indirectly on functionally connected brain areas relevant for tinnitus distress and tinnitus intensity in tinnitus patients. For rTMS studies, more specifically theta burst transcranial stimulation (TBS), only a few resting-state studies have explored whether TBS would lead to changes in resting-state functional connectivity in healthy subjects. The specific brain regions of interest encompassed a broad range including: (1) the occipital cortex (Rahnev et al., 2013); (2) anterior insula/frontal operculum (Gratton et al., 2013); (3) the primary somatosensory cortex (Valchev et al., 2015); (4) the primary motor cortex (Cárdenas-Morales et al., 2014; Cocchi et al., 2015; Noh et al., 2015); (5) the precuneus (Mancini et al., 2017); and (6) the DLPFC (Gratton et al., 2013; Mastropasqua et al., 2014; Iwabuchi et al., 2017). These studies all combined TBS with either resting-state EEG or resting-state fMRI to investigate whether TBS can induce increased or decreased functional connectivity between specific brain areas (e.g., intrahemispheric or interhemispheric connectivity changes; e.g., Rahnev et al., 2013; Noh et al., 2015; Valchev et al., 2015; Mancini et al., 2017) in healthy subjects. For example, studies targeting the left DLPFC in healthy subjects using TBS have demonstrated influence on regional and more remote functions using resting-state fMRI (e.g., Gratton et al., 2013; Iwabuchi et al., 2017). Gratton et al. (2013) demonstrated that left DLPFC cTBS increased connectivity with regions in the frontal, parietal, and cingulate cortices, suggesting that acute disruption by TBS to cognitive control regions can cause widespread changes in network connectivity not limited to the targeted networks. Iwabuchi et al. (2017) further explored whether prefrontal iTBS targeting the left DLPFC can modulate crucial limbic structures such as the insula, which can explain the therapeutic effects of DLPFC rTMS in depression. They have found that iTBS significantly dampened fronto-insular connectivity, demonstrating that left DLPF iTBS was able to modulate the right anterior insula (Iwabuchi et al., 2017).

Task-related neuroimaging studies have also examined short-term reorganization in healthy subjects (e.g., O’Shea et al., 2007; Andoh and Paus, 2011; Chib et al., 2013; Weber et al., 2014) after non-invasive neuromodulation. These studies all combined non-invasive neuromodulation such as tDCS or rTMS with neuroimaging such as EEG or fMRI, while performing a task, allowing for more process-specific findings. These studies demonstrate that non-invasive neuromodulation can modulate specific task functions, regions, and network interactions. Studies differ in the task performed, the neuromodulation technique used (e.g., tDCS or rTMS), the neuroimaging technique used (e.g., EEG or fMRI), and the specific brain region of interest. For example, Andoh and Paus (2011) combined off-line 10 Hz rTMS targeting the left and right posterior temporal area of Wernicke (LTMP; performed outside the magnetic resonance scanner) with fMRI, acquired during the performance of a word recognition task. Following the hypothesis that some brain functions operate in a state of interhemispheric compensation (i.e., recruiting homologous regions in the contralateral hemisphere) to recover after a virtual lesion (O’Shea et al., 2007), Andoh and Paus (2011) hypothesized that rTMS applied over the LTMP area will induce changes in the task-related fMRI response both locally and distally, namely in the homologous area in the contralateral hemisphere (RTMP). Similarly, they predicted that rTMS applied over the RTMP area would induce changes in the task-related fMRI response in the contralateral hemisphere (LTMP). Their result showed that rTMS increased task-related fMRI response in the homolog areas contralateral to the stimulated areas, consistent with the hypothesis regarding the role of homolog areas in the contralateral hemisphere for preserving behavior after neural interference. Interestingly, studies have also combined non-invasive neuromodulation with simultaneous task-based neuroimaging (e.g., Meinzer et al., 2012, 2013). For example, Meinzer and Colleagues have used fMRI during overt semantic word generation and simultaneous intrascanner anodal tDCS on the left inferior frontal gyrus (IFG) to investigate its effects on performance and task-related activity in healthy young adults (Meinzer et al., 2012) and in healthy older adults (Meinzer et al., 2013). The study in healthy young adults showed improved word-retrieval during anodal tDCS paralleled by selectively reduced task-related activation in the left ventral IFG, indicative of more efficient neural processing. In healthy older adults, anodal tDCS significantly improved performance up to the level of the younger controls and task-related fMRI showed reduced task-related hyperactivity in the bilateral prefrontal cortices, the anterior cingulate gyrus, and the precuneus (brain areas that were “hyperactive” in older compared to younger adults). Thus, this study showed that one single session of tDCS can temporarily reverse some age-associated changes in brain activity and connectivity.

### MICRO-Level: Assessing Network Connectivity With Chemical Markers of Neural Plasticity/Neurometabolites

While the induced effects of non-invasive neuromodulation have been investigated using neuroimaging techniques that provide information of the brain’s neural structure on a “macro” network-based connectivity level, tDCS studies have also been examining the effects of the brain’s neural structure on a “micro” neurometabolites level, in an attempt to explain local and distributed processing in functional brain networks (Hunter et al., 2013). TDCS has been known to influence neurophysiological mechanisms responsible for neuroplasticity by modulating the excitability of glutamatergic pyramidal neurons in the underlying cortex (Radman et al., 2009). These mechanisms involve the potentiation of synaptic glutamatergic receptors (Liebetanz et al., 2002; Nitsche et al., 2005) and decreased neurotransmission of GABA (Nitsche et al., 2004; Stagg et al., 2009; Stagg and Nitsche, 2011; Hunter et al., 2015; for a review, see Medeiros et al., 2012). Since there is evidence that the sum of glutamate and glutamine (Glx) levels are increased by tDCS (Clark et al., 2011) and that there is a strong relationship between Glx and functional connectivity (Horn et al., 2010; Kapogiannis et al., 2013), studies have been investigating whether tDCS-evoked changes in Glx may predict variations in functional connectivity within and between both local and distributed brain networks (e.g., Hunter et al., 2015). Hunter et al. (2015) found evidence that the after-effects of right parietal tDCS on glutamatergic signaling and network connectivity contribute to local, cross-hemispheric, and subcortical alterations. They found increases in baseline glutamatergic signaling that originate from the parietal site of the stimulation, with the precuneus possibly acting as an intermediate node that modulates glutamatergic signaling in other pathways that include the bilateral superior parietal lobule, ACC, salience, left frontal-parietal network, and the basal ganglia networks. They further suggest that cross-hemispheric connectivity, specifically for the bilateral inferior parietal network, may be more readily influenced by other neurotransmitter pathways, such as GABA. Recently, studies have been conducted to investigate the involvement of GABAergic signaling pathways that may influence network connectivity. Stagg et al. (2014) showed that tDCS on M1 decreased GABA levels within M1 and increased resting motor network connectivity. They suggested that the network-level connectivity within the motor system is related to the degree of inhibition in M1, a major node within the motor network. Bachtiar et al. (2015) replicated this tDCS experiment and confirmed the decreased GABA levels within M1 and increased resting motor network connectivity. However, they did not find a relationship between the change in GABA levels in M1 and the change in functional connectivity, suggesting that it might be driven by distinct underlying mechanisms.

## Various Stimulation Targets for a Specific Disorder

Non-invasive neuromodulation studies targeting different brain areas to treat one specific disorder have shown to have similar positive results. In this section, we provide support to demonstrate that rTMS and tDCS targeting different nodes of the dysfunctional brain network of a specific disorder can have similar positive effects, using tinnitus and depression as illustrative examples. See Tables 1, 2 for a summary of non-invasive brain stimulation (NIBS) targets for tinnitus and depression.

**Table 1:** Non-invasive brain stimulation (NIBS) targets in tinnitus.

Stimulation target	References
**Repetitive transcranial magnetic stimulation**
HF rTMS to the left auditory cortex	e.g., Plewnia et al. (2003) and Fregni et al. (2006)
LF rTMS to the left auditory cortex	e.g., Eichhammer et al. (2003) and Langguth et al. (2003)
LF to the right DLPFC	e.g., De Ridder et al. (2013)
Combined modulation of HF left DLPFC then LF left temporal cortex	e.g., Kleinjung et al. (2008)
Combined modulation of HF left DLPFC then LF left and right temporoparietal cortex	e.g., Lehner et al. (2013)
**Transcranial direct current stimulation**
Anode left auditory cortex—cathode contralateral supraorbital region	e.g., Fregni et al. (2006), Garin et al. (2011), Shekhawat et al. (2013) and Forogh et al. (2016)
Anode right DLPFC—cathode right DLPFC	e.g., Vanneste et al. (2010), Vanneste and De Ridder (2011), De Ridder and Vanneste (2012), Faber et al. (2012) and Frank et al. (2012)
Simultaneuos 1 anode prefrontal cortex—2 cathode left and right auditory cortex	e.g., Pal et al. (2015)

*HF, high frequency; rTMS, repetitive Transcranial Magnetic Stimulation; LF, low frequency; DLPFC, dorsolateral prefrontal cortex*.

**Table 2:** NIBS targets in depression.

Stimulation target	References
**Repetitive transcranial magnetic stimulation**
HF rTMS to left DLPFC	e.g., Pascual-Leone et al. (1996), Berman et al. (2000), Rossini et al. (2005), Loo et al. (2007), Bretlau et al. (2008), George et al. (2010) and Baeken et al. (2013, 2014)
LF rTMS to the right DLPFC	e.g., Klein et al. (1999), Januel et al. (2006), Fitzgerald et al. (2008), Bares et al. (2009) and Aguirre et al. (2011)
Combined modulation of HF left DLPFC then LF right DLPFC during same session	e.g., Hausmann et al. (2004), Fitzgerald et al. (2006a, 2012), Garcia-Toro et al. (2006), McDonald et al. (2006), Pallanti et al. (2010) and Blumberger et al. (2012a)
Combined modulation of HF left than right DMPFC	e.g., Downar et al. (2014), Salomons et al. (2014) and Bakker et al. (2015)
**Transcranial direct current stimulation**
Anode left DLPFC—cathode right supraorbital region	e.g., Boggio et al. (2008a,b), Loo et al. (2012), Palm et al. (2012) and
	Bennabi et al. (2015)
Anode left DLPFC—cathode right DLPFC	e.g., Ferrucci et al. (2009), Brunoni et al. (2013, 2014), Valiengo et al. (2013, 2016, 2017)

*HF, high frequency; rTMS, repetitive Transcranial Magnetic Stimulation; DLPFC, dorsolateral prefrontal cortex; LF, low frequency; DMPFC, dorsomedial prefrontal cortex*.

### Tinnitus

Tinnitus is a common and distressing disorder that is characterized by the perceived sensation of a sound in the absence of an external sound source (Langguth et al., 2012; De Ridder et al., 2014). Like other disorders, tinnitus can be perceived as a phenomenological unified coherent percept, binding separable clinical characteristics, such as the tinnitus loudness, the tinnitus sidedness, the tinnitus type (pure, tone, noise), the associated distress and so on (De Ridder et al., 2014). Based on neuroimaging studies, a group of tinnitus researchers have proposed a tinnitus brain model consisting of multiple parallel, dynamic, and partially overlapping subnetworks each representing a specific aspect of tinnitus (see De Ridder et al., 2014). However, the revealed subnetworks encoding the different aspects of the tinnitus percept, e.g., the distress network, can be similar to other pathologies, such as pain (Moisset and Bouhassira, 2007; De Ridder et al., 2013, 2014). Communication between these different subnetworks is proposed to occur at brain hubs, brain areas that are involved in multiple subnetworks simultaneously (De Ridder et al., 2014).

In non-invasive neuromodulation studies, researchers mostly targeted the left auditory cortex (temporal or temporoparietal cortical areas), but some have investigated the DLPFC as a target both in isolation and in a multisite stimulation approach, to suppress tinnitus. The left auditory cortex and the DLFPC are believed to be part of a neural network that appears to play a significant role in tinnitus perception (Shekhawat et al., 2016). The auditory cortex has become a common stimulation target for tinnitus, as past neuroimaging studies have shown over-activation of the left auditory cortex in tinnitus patients (Arnold et al., 1996; Lockwood et al., 1998). Repetitive TMS (Fregni et al., 2006), as well as anodal tDCS to the auditory cortex (left temporal or temporoparietal cortices areas; Fregni et al., 2006; Garin et al., 2011), were found to suppress tinnitus. For TMS, LF and high frequency (HF) were investigated to interrupt the tinnitus percept. The first rTMS case studies (sham-controlled) in tinnitus mostly investigated LF rTMS targeting the left auditory cortex in an attempt to inhibit the hyperactivity in this area (Eichhammer et al., 2003; Langguth et al., 2003). Eichhammer et al. (2003) found considerable improvement in tinnitus in two out of three patients after 1 week (five consecutive days) with LF (1 Hz) rTMS with 2000 stimuli per day, measured by the Tinnitus Questionnaire (assessing different tinnitus complaints e.g., distress, auditory perceptual difficulties, sleep disturbance; Eichhammer et al., 2003). Langguth et al. (2003) described a 4-week case study involving LF (1 Hz) rTMS treatment (5 days of treatment per week with 2000 stimuli/day) targeting the left auditory cortex. This treatment resulted in remarkable effects, enduring for several weeks, measured by the Tinnitus Questionnaire (assessing different tinnitus complaints e.g., distress, auditory perceptual difficulties, sleep disturbance) and paralleled by altered cortical excitability. Later on, HF rTMS was applied to the left auditory cortex with the belief that a virtual temporary lesion of this area can induce a transient reduction in tinnitus (Plewnia et al., 2003). Plewnia et al. (2003) applied HF (10 Hz) via five stimulation trains at different scalp positions in 14 tinnitus patients and found only a significant reduction in tinnitus when targeting the left temporal and left temporoparietal cortex (targeting the DLPFC did not yield in significant results) using a self-rating tinnitus reduction scale. Fregni et al. (2006) replicated the findings of Plewnia et al. (2003) applying HF (10 Hz) via nine stimulation trains of 30 stimuli and found transient tinnitus reduction, measured by the same self-rating tinnitus reduction scale, as well as a self-rating tinnitus intensity scale (Fregni et al., 2006). Moreover, they tested whether tDCS targeting the left auditory cortex would yield in similar effects. Their results showed that even 3 min of 1 mA anodal tDCS targeting the left auditory cortex induces similar transient tinnitus reduction as with HF rTMS (Fregni et al., 2006). TDCS, as well as other transcranial electrical stimulation (tES) techniques, such as transcranial random noise stimulation (tRNS) and transcranial alternating current stimulation (tACS), targeting the left auditory cortex has been further investigated by other researchers. Garin et al. (2011) showed that a single session 20-min session of 1 mA anodal tDCS significantly reduced tinnitus intensity, as measured by a Visual Analogue Scale. Although, Shekhawat et al. (2013) have demonstrated that 2 mA anodal tDCS for 20 min targeting the left auditory cortex was the most effective stimulation parameter for one session (measured by the Clinical Global Improvement measure and the Tinnitus Loudness Visual Analogue Scale), Forogh et al. (2016) did not find any significant effects when they applied the same for five sessions in patients with tinnitus (measured by the Clinical Global Improvement measure, the Tinnitus loudness Visual Analogue Scale, Tinnitus related-distress Visual Analogue Scale, and the Tinnitus Handicap Inventory). Interestingly, electrical stimulation where the stimulation current is varied randomly, i.e., tRNS was found to be superior both to tDCS (where stimulation current is held constant) and tACS (where stimulation current is time dependent with a sinusoidal shape) when applied bilaterally over the temporal cortex (Vanneste et al., 2013a; Van Doren et al., 2014). More recently, the introduction of High-Definition tDCS (HD-tDCS) has improved the spatial accuracy of conventional tDCS by using arrays of smaller “high-definition” electrodes instead of two large pad electrodes (Datta et al., 2009; Dmochowski et al., 2011; Guleyupoglu et al., 2013; Villamar et al., 2013a; Heimrath et al., 2015; Shekhawat et al., 2016). Shekhawat et al. (2016) have investigated the effect of stimulation location (left auditory cortex or DLPFC), stimulation duration (10 min or 20 min), and stimulation intensity (center anode 1 mA or 2 mA) on tinnitus loudness and annoyance using 4 × 1 HD-tDCS (Shekhawat et al., 2016). They concluded that a higher intensity (2 mA) and a longer duration (20 min) of stimulation were more effective, but either stimulation location was equally effective for suppressing tinnitus loudness and annoyance (Shekhawat et al., 2016).

The other common stimulation target investigated for transient tinnitus suppression is the DLPFC. The prefrontal cortex is believed to play a vital role in tinnitus, since it is critically involved in the integration of sensory and emotional aspects of tinnitus, as first mentioned by Jastreboff (1990; Kleinjung et al., 2008) and confirmed by recent studies as an essential part of the tinnitus distress network (Vanneste et al., 2010). Additionally, electrophysiological studies suggest that tinnitus occurs as a result of dysfunctional top-down inhibitory mechanisms originating in the prefrontal lobe (Norena et al., 1999), indicating that the prefrontal cortex does not only color tinnitus perception, but may efficiently switch the perceived signal on and off (Rauschecker et al., 2010; Frank et al., 2012), therefore being able to influence both tinnitus distress and intensity. Bifrontal tDCS where the anode is placed over the right DLPFC and the cathode over the left DLPFC, has been investigated in several tinnitus studies (Vanneste et al., 2010; Vanneste and De Ridder, 2011; De Ridder and Vanneste, 2012). The first clinical study conducted in 478 tinnitus patients by Vanneste et al. (2010) revealed that one 20-min session of 1.5 mA tDCS with a right anode and left cathode set up (and not left anode, right cathode) modulated tinnitus perception in 29.9% of the tinnitus patients. For these responders, a significant reduction was found for both tinnitus intensity and tinnitus-related distress (Vanneste et al., 2010). These results were further confirmed in a follow up study in which resting-state EEG was added before and after tDCS to unravel the mechanism by which tDCS suppresses tinnitus (Vanneste and De Ridder, 2011). This study provides evidence that bifrontal tDCS can suppress tinnitus intensity and tinnitus-related distress by modulating the pregenual ACC, the parahippocampal area, and the right primary auditory cortex in resting-state spontaneous brain activity (Vanneste and De Ridder, 2011). Furthermore, the study by De Ridder and Vanneste (2012) also supports bifrontal tDCS as a technique to reduce tinnitus intensity and distress, as one session of bifrontal tDCS elicited a larger reduction in tinnitus intensity and distress compared to EEG driven tDCS. TACS targeting the DLPFC has also been investigated (Vanneste et al., 2013b), as it could theoretically normalize alpha power, which is known to decrease in tinnitus (Lorenz et al., 2009). However, tACS did not modulate tinnitus loudness and annoyance like bifrontal tDCS was able to (Vanneste et al., 2013b). Therefore, repetitive sessions of bifrontal tDCS was investigated as a potential treatment (De Ridder and Vanneste, 2012; Faber et al., 2012; Frank et al., 2012). Frank et al. (2012) found that six 30-min sessions of 1.5 mA tDCS (right anode and left cathode) only resulted in a small clinical impact on tinnitus loudness and discomfort. Faber et al. (2012) investigated six sessions of tDCS for left and right anodal DLPFC and found that both active conditions (irrespective of the anodal position) were able to reduce tinnitus annoyance but not tinnitus intensity. For magnetic stimulation, LF (1 Hz) rTMS on the right DLPFC also resulted in a reduction in the perceived loudness of tinnitus after one session (200 pulses), as well as after 10 sessions of rTMS (600 pulses), which is mediated by the functional connections between the DLPFC, and a network consisting of the ACC, the parahippocampus, and the auditory cortex (De Ridder et al., 2013).

An increasing amount of studies are investigating the stimulation the two common stimulation targets in tinnitus patients, the auditory cortex and the DLPFC, simultaneously or one stimulation after the other. One tDCS study simultaneously targeted the auditory cortex with the DLPFC by placing the cathode on the auditory cortex and the anode over the prefrontal cortex for five sessions (Pal et al., 2015). However, this stimulation set up did not yield in an improvement in any tinnitus measures (Pal et al., 2015). Two studies suggest that the efficacy of rTMS in tinnitus can be enhanced by stimulating prefrontal cortical areas in addition to the auditory cortex (Kleinjung et al., 2008; Lehner et al., 2013). A study by Kleinjung et al. (2008) combined HF prefrontal and LF temporal rTMS. Patients received either LF temporal rTMS or a combination of HF prefrontal and LF temporal rTMS. Directly after therapy they found an improvement for both groups, but no differences between the groups. An evaluation after 3 months revealed a remarkable benefit from the use of combined prefrontal and temporal rTMS treatment. These findings suggest that auditory and non-auditory brain areas are involved in tinnitus psychopathology. Another study by Lehner et al. (2013) combined HF prefrontal with LF left and right temporal rTMS (left DLPFC, left temporoparietal cortex, and right temporoparietal cortex). Patients received either LF temporal rTMS or a combination of HF prefrontal and LF temporal rTMS. They found that multisite rTMS is significantly superior to temporal rTMS and represents a promising strategy for enhancing treatment effects of rTMS in tinnitus. Several authors have (e.g., Vanneste and De Ridder, 2012) already stated that the perception of tinnitus involves large and complex interconnected networks and that tinnitus can be a result of a dysfunction in any part of the system. Thus, modulation of any part of this network may interfere with tinnitus perception (Vanneste and De Ridder, 2012). However, stimulating the auditory cortex alone might not be sufficient to achieve long lasting improvement of tinnitus severity (Lehner et al., 2013). Accordingly, the idea of network stimulation as a new stimulation protocol is promising and needs to be further investigated.

### Depression

Depression is clinical disorder known to result from a disruption of brain neurochemistry (Akhtar et al., 2016). It is a neuronal abnormality characterized by disorders of mood, cognitive function, and neurovegatative functions and has a wide range of causes (Akhtar et al., 2016). Previous neuroimaging studies have demonstrated structural and functional abnormalities in distributed networks of cortical and limbic brain regions including the DLPFC, ventromedial prefrontal cortex, amygdala, hippocampus and subgenual cingulate amongst others (Campbell et al., 2004; Mayberg, 2007; Drevets et al., 2008; Koenigs and Grafman, 2009). Structural and functional abnormalities in these brain regions have also been suggested to be associated with negative affective and cognitive processing bias of depressed patients (Liu S. et al., 2017). Studies have suggested that during the affective processing of depressive individuals, network abnormalities, manifested as enhanced affective processing and decreased cognitive control function, might lead to a more intense experience of negative emotion, inducing depression (Liu S. et al., 2017).

In non-invasive neuromodulation studies, researchers have mostly targeted the left and right DLPFC for depression, as conventional tDCS or traditional rTMS coils were not able to directly and selectively target deeper limbic regions such as the subgenual ACC (Fox et al., 2012a). Deeper limbic regions such as the subgenual ACC have been targeted with invasive neuromodulation, such as deep brain stimulation (e.g., Mayberg et al., 2005; Drevets et al., 2008; Mayberg, 2009; Fox et al., 2012a) to decrease hyperactivity in this brain region as this has been associated with antidepressant response in depressed patients (Mayberg et al., 2005; Drevets et al., 2008). Regarding more superficial brain areas, researchers have mostly targeted the left DLPFC, but the right DLPFC has also been investigated both in isolation and in a multisite stimulation approach, to suppress depression. The left DLPFC has been found to be hypoactive in patients with depression and an increase in activity is associated with antidepressant response (Fitzgerald et al., 2006b; Koenigs and Grafman, 2009). Besides the hypoactivity found in the left DLPFC, neuroimaging studies have also demonstrated hyperactivity in the right DLPFC in depressive disorder (Bench et al., 1995; Lefaucheur et al., 2014). One of the first FDA-approved clinical uses of rTMS is HF stimulation to the left DLPFC for the treatment of depression (George et al., 1995; Pascual-Leone et al., 1996; O’Reardon et al., 2007; Padberg and George, 2009). In general, the efficacy of HF rTMS to the left DLPFC using a variety of parameters has been established (For review, see Lefaucheur et al., 2014; e.g., Pascual-Leone et al., 1996; Berman et al., 2000; Rossini et al., 2005; Loo et al., 2007; Bretlau et al., 2008; George et al., 2010; Baeken et al., 2013, 2014). LF rTMS to the right DLPFC has also been found effective in several studies (for a review, see Lefaucheur et al., 2014; e.g., Klein et al., 1999; Januel et al., 2006; Fitzgerald et al., 2008; Bares et al., 2009; Aguirre et al., 2011) and there seems to be no difference in antidepressant effect between HF of the left DLPFC and LF rTMS of the right DLPFC (Lefaucheur et al., 2014). For tDCS, earlier studies have mostly targeted the left DLPFC using the electrode montage where the anode is placed over the left DLPFC and the cathode over the right orbitofrontal cortex (for a review, see Lefaucheur et al., 2017; e.g., Boggio et al., 2008a; Loo et al., 2012; Palm et al., 2012; Bennabi et al., 2015). More recently, studies have applied bilateral tDCS, placing the anode electrode over the left DLPFC and the cathode over the right DLPFC instead of the right supraorbital region (e.g., Ferrucci et al., 2009; Brunoni et al., 2013, 2014; Valiengo et al., 2013, 2016, 2017). Several rTMS studies have also investigated a combined modulation of HF left DLPFC rTMS and LF right DLPFC rTMS during the same sessions in the same patients (e.g., Hausmann et al., 2004; Fitzgerald et al., 2006a; Garcia-Toro et al., 2006; McDonald et al., 2006; Pallanti et al., 2010; Blumberger et al., 2012a; Fitzgerald et al., 2012). Lefaucheur et al. (2014), however, do not recommend of using this bilateral rTMS, because of contradictory results across studies.

NIBS has been proposed to normalize the interhemispheric imbalance of neuronal activity by excitatory stimulation of the left DLPFC and inhibitory stimulation over the right DLPFC (Palm et al., 2016; Lefaucheur et al., 2017). According to the “valence hypothesis” the affective processing exhibits hemispheric lateralization, with the right hemisphere specializing in negative emotional processing and the left hemisphere specializing in positive emotional processing (Prete et al., 2015; Liu S. et al., 2017). To further investigate how rTMS of the DLPFC exerts its antidepressant effect, Fox et al. (2012a) have investigated why some left DLPFC rTMS targets are more effective than others by examining differences in functional connectivity of these sites to deeper limbic regions, using resting-state fMRI. They have found that the DLPFC TMS sites with better clinical efficacy were negatively correlated (anticorrelated) with the subgenual ACC. This might suggest a role for intrinsically anticorrelated brain networks in depression implying that the clinical efficacy of focal brain stimulation could be optimized by targeting based on connectivity (Fox et al., 2012a). Depression has indeed been associated by altered intrinsic functional connectivity within and between three intrinsic connectivity networks (ICNs), such as the DMN, the central executive network (CEN) and the salience network (SN; Manoliu et al., 2013; Anderson et al., 2016; Liu S. et al., 2017). Abnormally increased DMN connectivity has been found in association with depression (Anand et al., 2005; Greicius et al., 2007; Broyd et al., 2009; Sheline et al., 2010; Liston et al., 2014; Kaiser et al., 2015), more specifically related to rumination and deficits in emotion regulation (Sheline et al., 2009; Hamilton et al., 2011). The CEN or the task-positive network, with the DLPFC as a one of the primary nodes, has been found to be hypoconnected in patients with depression (Liston et al., 2014; Kaiser et al., 2015), which may contribute to deficits in memory and attention and other cognitive symptoms in depression (Liston et al., 2014). Furthermore, the SN has shown decreased connectivity in patients with depression, with this aberrant connectivity also demonstrating a significant relationship to depressive symptom severity (Manoliu et al., 2013). Furthermore, these large-scale functional networks have been found to be interacting with each other, with dysfunction in these dynamics associated with depressive symptomatology (Menon, 2011; Manoliu et al., 2013; Anderson et al., 2016). For instance, in depression unusual connectivity has been reported between networks, including abnormal hyperconnectivity between the SN and DMN, and between the DMN and CEN (Manoliu et al., 2013; Liston et al., 2014; Kaiser et al., 2015). In depressed individuals a dominance of DMN over CEN was found which correlated with maladaptive rumination (Hamilton et al., 2011; Wang et al., 2016). Moreover, the aberrant switching between the DMN and CEN in depression has been suggested as a mechanism underlying the preoccupation with self-referential processes related to DMN hyperactivity, and deficits in cognitive functioning, associated with CEN hypoconnectivity (Menon, 2011; Manoliu et al., 2013; Kaiser et al., 2015; Anderson et al., 2016; Wang et al., 2016). The SN has been found to play a causal role in mediating this switching between the DMN and CEN activity (Wang et al., 2016), with specifically the right anterior insula associated with the aberrant DMN/CEN interactions and severity of depressive symptoms (Manoliu et al., 2013). Overall, this evidence demonstrates that dysfunction in connectivity within large-scale functional networks and the interactions in connectivity between these networks are associated with depression (Anderson et al., 2016). Moreover, Liston et al. (2014) have examined the effect of non-invasive neuromodulation in affecting these large-scale brain networks and their relation to treatment response using HF (10 Hz) TMS targeting the left DLPFC during a 5-week period (25 sessions). The treatment normalized the depression-related subgenual hyperconnectivity in the DMN, but did not alter the diminished connectivity in the CEN (Liston et al., 2014). TMS also induced an anticorrelated connectivity between the DLFPC and the medial prefrontal DMN nodes (Liston et al., 2014).

Another brain region that has been considered a key region in emotion regulation (Mayberg et al., 1999) and that has recently been investigated as a new rTMS target for depression is the dorsomedial prefrontal cortex (DMPFC; i.e., DMN node; Downar and Daskalakis, 2013; Anderson et al., 2016; Liu S. et al., 2017; Liu W. et al., 2017). Studies have suggested that rTMS targeting the DMPFC could show similar antidepressant findings in patients with depression and is a safe procedure (e.g., Downar et al., 2014; Salomons et al., 2014; Bakker et al., 2015). HF rTMS applied bilaterally over the DMPFC demonstrated increased anticorrelation between the DMPFC (DMN node) and the insula (SN node), and increased connectivity with the thalamus (CEN node) to be associated with a greater clinical response in patients with depression (Salomons et al., 2014; Anderson et al., 2016). However more research is needed to investigate the DMPFC as a target for clinical application of rTMS in depression (Liu S. et al., 2017).

## One Stimulation Target for Different Disorders

Non-invasive neuromodulation studies stimulating one specific brain region have demonstrated efficacy for various disorders. In this section, we gather evidence to show that tDCS and rTMS over an important brain hub involved in different processes and disorders, i.e., the DLPFC, can have an effect on healthy subjects as well as on various patient populations. The DLPFC is an important brain hub for general cognitive and emotional processing and stimulation of this brain area is expected to influence different processes and disorders.

The DLPFC has been the most common stimulation target in clinical literature to date (Downar et al., 2016). The DLPFC has been the target in several tDCS studies. Anodal tDCS to the left DLPFC has been studied in healthy subjects as well in patient populations. Studies in healthy subjects targeting the DLPFC using anodal tDCS have reported to transiently improve working memory (e.g., Fregni et al., 2005) and attention in healthy subjects (e.g., Nelson et al., 2014). In patient populations, tDCS targeting the DLPFC has been found to show transient improvement in attention in patients with traumatic brain injury (e.g., Kang et al., 2012), in working memory in patients with Parkinson’s disease (e.g., Boggio et al., 2006), in recognition memory in patients with Alzheimer disease (e.g., Ferrucci et al., 2008), in signs of consciousness in patients in a minimally conscious state (e.g., Angelakis et al., 2014; Thibaut et al., 2014, 2017), in mood in patients with depression (e.g., Blumberger et al., 2012b; Loo et al., 2012; Palm et al., 2012), in auditory hallucinations in patients with schizophrenia (e.g., Brunelin et al., 2012) and in craving in substance abusers (e.g., Boggio et al., 2008b, 2009; Fregni et al., 2008). In rTMS studies, the left and right DLPFC have been the target for LF and HF rTMS studies in depression (e.g., George et al., 2010; Triggs et al., 2010; Ray et al., 2011; Baeken et al., 2013, 2014), post-traumatic stress disorder (e.g., Boggio et al., 2010; Watts et al., 2012), panic disorder (e.g., Mantovani et al., 2013), schizophrenia targeting the negative symptoms (e.g., Rollnik et al., 2000; Hajak et al., 2004; Cordes et al., 2010; Prikryl et al., 2013) and in addiction disorders—more specifically food (e.g., Van den Eynde et al., 2010; Barth et al., 2011), alcohol (e.g., Mishra et al., 2010) and cigarettes (e.g., Li et al., 2013; Prikryl et al., 2014).

These studies support the idea that neuromodulation of important brain hubs such as the DLPFC can have an effect on different processes in healthy subjects and on different disorders through its influence on a functional brain network. In patient populations, studies have associated the (dorsolateral) prefrontal cortex with top-down prefrontal control, important in various disorder including tinnitus (Mitchell et al., 2005), substance use disorder (Bradshaw et al., 2017), pain (Martinez et al., 2017), depression (Ochsner and Gross, 2005) and schizophrenia (Mayer et al., 2015). A recent study by Tik et al. (2017) further demonstrated that stimulation of the left DLFPC modulates ACC connectivity in a specific meso-cortico-limbic network, which might explain the treatment mechanism of psychiatric disorders such as depression.

In healthy subjects, studies have focused on cognitive processing. Tremblay et al. (2014) have concluded that prefrontal tDCS targeting the DLFPC has the potential to modulate numerous cognitive functions simultaneously and that the effect of prefrontal tDCS on a given task is probably associated with the extensive modulation of a wide range of cognitive functions. Using resting state functional connectivity, Krishnamurthy et al. (2015) further suggest that tDCS might prime not just the underlying neocortex, but an extended network that can be recruited according to the task demands. According to the authors, these priming effects might explain why similar montages have yielded tDCS-induced effects across multiple motor and cognitive tasks. Luft et al. (2014) suggest that the DLPFC can be considered a functional “flexible hub”. A flexible hub is defined as a brain region that rapidly updates its pattern of global functional connectivity according to the task demands (Luft et al., 2014). These flexible hubs play an important role in switching from one state to the other in order to attend to the necessary task demands (Luft et al., 2014). Thus, there are indications that modulating an important brain hub using neuromodulation can influence a whole brain network.

## Discussion

The brain is a complex network and, therefore, studying and treating brain disorders using non-invasive neuromodulation techniques should be approached as a network phenomenon. In this article, we have presented support from different perspectives to demonstrate that non-invasive neuromodulation techniques, such as rTMS and tDCS modulate brain networks rather than just local stimulation targets. Evidence for the effectiveness of different stimulation targets for one disorder as well as evidence for the effectiveness of one stimulation target for different disorders indicates an underlying neural network for disorders and, thus, points to the idea of network stimulation as a novel stimulation protocol. Moreover, studies assessing network connectivity on both the macro and micro levels help describe and explain the distributed processing of neuromodulation in functional brain networks. However, there are some limitations to connectivity assessed with NIBS in a clinical context. First, NIBS, e.g., tDCS and rTMS, stimulates neuronal tissues exogenously and artificially and, thus, connectivity revealed by stimulation may be different than connectivity present under more physiological conditions (Fox et al., 2012b). Also, connectivity measured with rTMS alone can only be assessed in the cortex with a clear output effect, such as the motor or the visual cortex; investigating other brain areas or the connectivity between other structures needs the addition of neuroimaging techniques. For tDCS, as the electrical currents delivered by tDCS are not strong enough to fire an action potential, connectivity can only be assessed using neuroimaging techniques. Further, non-invasive measurements of human brain activity are highly susceptible to noise and remote changes observed in response to NIBS by neuroimaging techniques and could reflect other factors besides propagation of NIBS activity along the cortical connection creating interpretative ambiguity (Fox et al., 2012b). These factors could include behavioral and cognitive consequences of NIBS leading to changes in brain activity, or neural adaptation to the NIBS (Fox et al., 2012b). Combining non-invasive neuromodulation with neuroimaging and brain network theories will further elucidate the impact of neuromodulation on brain connectivity and will assist in the development of stimulation protocols to target brain networks, and not just brain regions.

## Author Contributions

WTT prepared the manuscript draft with important intellectual input from DDR, JH and SV. All authors approved the final manuscript.

## Conflict of Interest Statement

The authors declare that the research was conducted in the absence of any commercial or financial relationships that could be construed as a potential conflict of interest.
